# Parental Investment by Birth Fathers and Stepfathers: Roles of Mating Effort and Childhood Co-residence Duration

**DOI:** 10.1007/s12110-023-09450-6

**Published:** 2023-06-10

**Authors:** Jenni E. Pettay, Mirkka Danielsbacka, Samuli Helle, Gretchen Perry, Martin Daly, Antti O. Tanskanen

**Affiliations:** 1 Department of Social Research, University of Turku, Assistentinkatu 7, 20014 Turku, Finland; 2 Population Research Institute, Helsinki, Finland; 3 Department of Sociology, Anthropology & Human Services, University of Canterbury, Christchurch, New Zealand; 4 Department of Psychology, Neuroscience & Behaviour, McMaster University, Hamilton, ON Canada

**Keywords:** Family, Kinship, Intergenerational relations, Parenting, Father-child relations

## Abstract

This study investigates the determinants of paternal investment by birth fathers and stepfathers. Inclusive fitness theory predicts higher parental investment in birth children than stepchildren, and this has consistently been found in previous studies. Here we investigate whether paternal investment varies with childhood co-residence duration and differs between stepfathers and divorced birth fathers by comparing the investment of (1) stepfathers, (2) birth fathers who are separated from the child’s mother, and (3) birth fathers who still are in a relationship with her. Path analysis was conducted using cross-sectional data from adolescents and younger adults (aged 17–19, 27–29, and 37–39 years) from the German Family Panel (pairfam), collected in 2010–2011 (*n* = 8326). As proxies of paternal investment, we used financial and practical help, emotional support, intimacy, and emotional closeness, as reported by the children. We found that birth fathers who were still in a relationship with the mother invested the most, and stepfathers invested the least. Furthermore, the investment of both separated fathers and stepfathers increased with the duration of co-residence with the child. However, in the case of financial help and intimacy, the effect of childhood co-residence duration was stronger in stepfathers than in separated fathers. Our findings support inclusive fitness theory and mating effort theory in explaining social behavior and family dynamics in this population. Furthermore, social environment, such as childhood co-residence was associated with paternal investment.

**Supplementary Information:**

The online version contains supplementary material available at 10.1007/s12110-023-09450-6.

Between 1960 and 2010, rates of childbirth outside marriage, marital instability, and stepfamily formation increased substantially in most European countries, including Germany (Thomson, 2014). Nevertheless, the current prevalence of step families is not unprecedented. Studies of both historical and agricultural populations from Europe (Pettay et al., 2020; Voland & Willführ, 2017; Warner & Erdélyi, 2022) and contemporary hunter-gatherers (Bentley & Mace, 2009; Gray & Anderson, 2010) suggest that ancestral societies often matched or even surpassed contemporary ones with respect to the diversity and complexity of family structure. Human parental investment has therefore likely evolved to vary adaptively with complex family compositions (Sear, 2016). How important is fathers’ investment in humans? Although paternal investment is not necessary for infant survival in many societies (Sear & Mace, 2008), paternal investment may substantially improve children’s socioeconomic status and thus also provide fitness benefits (Geary, 2000; Sear & Mace, 2008).

Parental investment (Trivers, 1972) is defined as the allocation of resources in such a way as to benefit one or more of one’s offspring at the expense of one’s capacity to invest in other offspring. Hamilton (1964) expanded the fitness concept to encompass expected genetic posterity through one’s effects on both descendant and collateral kin (“inclusive fitness”), and this is the quantity that motives and emotions presumably evolve to maximize. Investment in unrelated stepchildren thus presents a puzzle for evolutionists. Although a man might invest in an unrelated child as a result of misattributed paternity, a typical stepfather has entered into stepfatherhood knowing that he is not a genetic father.

The dominant evolutionary explanation for stepfathering is that it functions as *mating effort*, meaning that the investment is in the new partnership with the child’s mother rather than in the child itself (Gray & Anderson, 2010). This interpretation is supported by comparative evidence: stepparenting is observed in some animal species with biparental care, and it is apparently confined to species that form enduring pairs such that a successful courtship can have long-term benefits (Rohwer, 1986; Rohwer et al., 1999). Paternal investment may also function as mating effort, but unlike stepfathering, it has direct fitness benefits as well (Anderson et al., 1999a). In humans, the inclination to invest in a relationship partner is not necessarily dependent on reproductive potential, as for example when men court and invest in postreproductive female partners (Anderson et al., 1999a).

Relative to birth fathers, stepfathers tend to invest less in children (e.g.,Anderson et al., 1999a; Flinn, 1988; Marlowe, 1999; Tooley et al., 2006) and, albeit rare, to assault, exploit, and kill them at higher rates (Daly, 2022; Daly & Wilson, 2008). On average, living with a stepparent is associated with various negative educational, cognitive, emotional, and behavioral outcomes compared with living in an intact two-parent family from birth (Sweeney, 2010). In historical and agricultural populations from Europe, in which children acquired stepfathers primarily after the father’s death rather than after divorce, a widowed mother’s remarriage often improved the family’s resources (Andersson et al., 1996; Pettay et al., 2014, 2020; Willführ & Gagnon, 2013), but it was also sometimes associated with elevated child mortality (Voland, 1988).

In contemporary Western societies, the acquisition of a stepfather is associated with improvements in children’s circumstances and prospects relative to those living with single mothers in some cases but not in others (Biblarz & Raftery, 1999; Erola & Jalovaara, 2017; Ferri, 1984; McLanahan & Sandefur, 1994). Although less research has been conducted in low-income societies, one such example comes from Serbian Roma communities, where growth and development indicators of young children did not differ in the presence of a father versus stepfather (Čvorović, 2022). Indeed, stepfather-stepchild relationships are often found to be positive, and many studies have found that stepfathers do invest in stepchildren. For example, although Dutch stepparents are less involved than birth parents, they still spend considerable time with their stepchildren, and co-residence is important for parental involvement (Arat et al., 2022).

Most research has focused on step relations in families with underage children, but a few studies have found that stepfathers continue to support adult stepchildren even after they have left their childhood home (Hornstra et al., 2020; Klaus et al., 2012). However, closeness to stepfathers does not always carry over into adulthood (King & Lindstrom, 2016). Clearly, there is a lot of variation among stepfathers in how much they invest in and how good a relationship they build with their stepchildren.

Several studies have found a positive association between the duration of co-residence between stepfather and child and the strength or quality of their relationship. For example, two studies from the Netherlands (Hornstra et al., 2020; Kalmijn, 2013) found that the longer a stepfather had resided with a child, the closer their relationship and the more frequent their contacts after the child had become independent. Daly et al. (1996) used parents’ ability to guess their undergraduate children’s opinions and attitudes as an index of parental interest and of parent–child closeness and found that, while stepfathers were significantly less accurate than birth fathers, their accuracy was an increasing function of the duration of their association with the child. As regards actual investment in children, however, evidence on the effects of co-residence duration is scarce. Anderson et al. (1999b) reported that it was an important predictor of stepfathers’ contributions to school expenditures for Xhosa adolescents in South Africa, but the same research team found no effect on self-reported investments by stepfathers in a US study (Anderson et al., 1999a). A Dutch study found that the longer a child had lived with a parent in their youth, the more types of support the parent provided the child in adulthood; support was measured as practical support, advice, support with child care, and financial support (van Houdt et al., 2019).

Childhood co-residence increases parents’ opportunities to invest in a child, fostering a stronger relationship and continued investment when the child reaches adolescence or adulthood (Ivanova & Kalmijn, 2020). This proximate idea of continued investment is compatible with an evolutionary explanation that individuals have evolved psychological mechanisms that lead them to preferentially invest in individuals cued as close kin (Rotkirch, 2018). Childhood co-residence is an important cue for kin detection, and co-residence may produce a feeling of kinship between the parties involved (Lieberman et al., 2007). When children and stepparents have lived with each other in the same household, they can form a “kin-like” bond and perceive themselves as emotional kin such that childhood co-residence can increase psychological attachment to stepchildren and increase investment from the stepparent (Rotkirch, 2018).

Does childhood co-residence duration affect investment by and relationship quality in birth fathers as well as in stepfathers? Kalmijn (2013) reported that co-residence duration was a predictor of adult children’s contact frequency and exchanges with both categories of fathers. Of course, the co-residence duration has a somewhat different meaning in the two cases: for stepfathers, a longer duration typically reflects an earlier child age at the start of co-residence, whereas for birth fathers, it typically reflects an older child age at its end, and the mother may play an important role in determining whether any father-child relationship continues. Regardless, childhood co-residence duration is a potentially important source of variation in contact, investment, and exchange between adult children and both their separated birth fathers and their stepfathers.

We suggest, however, that childhood co-residence may be more important for stepfathers than for birth parents because birth fathers have a fitness-based rationale for investing in their children after divorce, whereas stepfathers may need the “attachment boost” from close association during childhood in order to feel any inclination to invest. Indeed, a prior study found that lower closeness with stepfathers than birth fathers was partly explained by childhood co-residence and stepfathers’ involvement during youth (Ivanova & Kalmijn, 2020).

Here, we examine parental investment of birth fathers and stepfathers from an evolutionary perspective. Our study includes adolescent and adult children, which helps us to estimate the persistence of investment by stepfathers beyond the duration of childhood co-residence. We use cross-sectional data from the German Family Panel (pairfam), which is a suitable dataset for this study as it offers information on both childhood household composition and intergenerational relationships in later life (Huinink et al., 2011). Previously, analyzing data from pairfam, Arránz Becker et al. (2013) found that parents reported lower emotional closeness with their adolescent and adult stepchildren than birth offspring. They also considered the full duration of step relationships, and found that social interaction might mitigate the step gap. They did not, however, differentiate co-residence duration or investigate measures other than emotional closeness. We operationalized kin investment using financial and practical help, emotional support, intimacy, and emotional closeness. We hypothesized on the basis of previous research and inclusive fitness theory that:

- H1 Birth (genetic) fathers would invest more than stepfathers.
- H2 Birth fathers in a continuing relationship with the mother would invest more than those separated from her.
- H3 Duration of co-residence with the child would be positively associated with investment of both birth fathers and stepfathers.

However, because birth fathers have a fitness interest in the child regardless of whether they remain in a relationship with the mother, we further hypothesized that:

- H4 The association between co-residence duration and investment by separated birth fathers would be weaker than the same association among stepfathers.

## Data and Methods

We used survey data from the Panel Analysis of Intimate Relationships and Family Dynamics (pairfam), which offers information on intergenerational relations, childbearing, and several socioecological factors in Germany (Huinink et al., 2011). Pairfam provides longitudinal data on three birth cohorts born in 1971–1973, 1981–1983, and 1991–1993. We used wave 2 cross-sectional data conducted in 2010–2011, when the cohort members were approximately 17–19, 27–29, and 37–39 years of age; these data were used because questions concerning both childhood living arrangements and intergenerational relations were recorded in this wave.

### Measures

The parental investment variables used as dependent variables were from the intergenerational relations section of the pairfam questionnaire. Respondents answered questions about their relationships with parents with whom they still had contact. These parents include both biological parents (even after separation) and stepparents, when they existed. We investigate birth fathers and stepfathers. Dependent variables considered as proxies of investment here are financial help, practical help, intimacy, emotional support, and emotional closeness. While financial and practical help are concrete investments of money and time, emotional support is also an investment of time, and measures such as emotional closeness have been used as proxies for parental investment (e.g., Euler, 2011; Pollet & Hoben, 2011; Tanskanen et al., 2021).

*Financial help* is a composite (i.e., the mean) of two variables, which both measure material benefits (*r* = 0.48). Participants were asked: (1) how often they received financial help from the parent in question during the past 12 months and (2) how often they received gifts of money or valuables (more than 100 euros per gift) from the parent in question during the past 12 months; both variables had 5 categories, ranging from never = 0 to very often = 4. A second composite variable was the mean response to two questions about intimacy with parents (*r* = 0.66): (1) how often participants told their parents about what they were thinking and (2) how often they shared secrets and private feelings with them, both ranging from never = 0 to always = 4. Practical help was based on a question regarding how often the participant received help from the parent with shopping, housework, or yardwork during the past 12 months (never = 0 to very often = 4). Emotional support was based on a question regarding how often the parent in question talked to the respondent about the latter’s worries and troubles during the past 12 months (never = 0 to very often = 4), and Emotional closeness was based on a question regarding how close the respondent felt to the parent today (not at all close = 0 to very close = 4).

Childhood co-residence duration with birth father and stepfather were determined from questions about living arrangements before the age of 18 years. Shared physical custody is rare in Germany, and after a divorce children are much more likely to live with the mother than with the father (Walper et al., 2021). For birth fathers, co-residence duration was the number of years in which the respondent lived in the same household with him until the age of 18 or the age at which the survey was taken if the respondent was under 18. For analysis we divided birth fathers into two classes: those who were and those who were not in a relationship with the mother at the time of the interview. Separation from the mother could have happened any time during the childhood or after the respondent turned 18, so separated fathers could also have had full childhood co-residence duration with the respondent. We assumed that the last stepfather the respondent lived with during childhood would be the corresponding stepfather at the time of survey; hence, childhood co-residence duration was calculated as 18 years, or age of respondent if under 18, minus the age when the respondent started living with the stepfather. Co-residence duration for stepfathers ranged from 0 to 17 years, and for separated birth fathers from 0 to 18 years (see Tables 1 and 2 for descriptive statistics). Because we were comparing parental investment of stepfathers and birth fathers, respondents with adoptive parents were excluded (*N* = 50).

**Table 1:** Descriptive statistics of respondents

	No. of observations	%	Missing %
Birth cohort			0
1991–1993	3313	43.09	
1981–1983	2284	29.70	
1971–1973	2092	27.21	
Respondent’s gender			0
Male	3770	49.03	
Female	3919	50.97	
Respondent’s ethnicity			1.3
German native	5929	78.11	
Other countries	1662	21.89	
Respondent’s education			0.04
currently enrolled	2202	28.65	
primary and lower secondary	850	11.06	
upper secondary	2428	31.59	
post-secondary	849	11.05	
tertiary	1357	17.66	
Mother’s education			16.7
Lower level education	5048	78.88	
Higher level education	1352	21.13	
Respondent lives with mother			
No	4122	53.61	
Yes	3567	46.39	
Relationship status			0.7
not cohabiting	4762	62.38	
cohabiting	2872	37.62	
Children			0.1
no children	5417	70.51	
one or more child	2266	29.49

**Table 2:** Descriptive statistics of fathers and stepfathers

	Biological father			Separated father			Stepfather			Missing
	*n*	%/mean	SD	*n*	%/mean	SD	*n*	%/mean	SD	%
No. of observations	5420			1735			1171			
Financial help	5398	1.45	1.07	1722	1.09	1.04	1163	0.84	0.96	0.52
Practical help	5329	0.90	1.17	1708	0.51	0.99	1150	0.58	1.02	1.67
Emotional support	5394	1.33	0.99	1721	1.13	1.05	1161	0.84	1.00	0.60
Intimacy	5417	1.57	0.86	1734	1.37	0.96	1169	1.01	0.89	0.07
Emotional closeness	5414	2.98	0.92	1731	2.52	1.12	1169	2.18	1.07	0.14
Childhood co-residence duration	5420	17.42	1.38	1735	13.45	5.91	1171	3.27	5.11	
Travel time from respondent dwelling to (step) father’s house										3.50
live in the same house	3055	38.02		62	0.77		416	5.18		
less than 10 min	782	9.73		311	3.87		164	2.04		
10–30 min	545	6.78		373	4.64		195	2.43		
30–60 min	278	3.46		249	3.1		130	1.62		
1–3 h	289	3.6		202	2.51		113	1.41		
3 h or more	417	5.19		324	4.03		130	1.62		

Other variables included in the models, when available, were gender (two levels: male and female), cohort (three levels: 1971–1973, 1981–1983, and 1991–1993), ethnicity based on parent’s birth country (two levels: native German and other countries), mother’s education (based on ISCED-97 classification and grouped in two levels: higher [tertiary] and lower [secondary or lower] education), respondent’s education (continuous based on ISCED-97 classification: ranging from still enrolled to tertiary education), cohabiting status (cohabiting or not co-habiting with someone), children (two levels: none, or one or more biological children alive), travel time from respondent dwelling to father/stepfather’s house (continuous: ranging 0 = living in the same household to three hours or more), and living with mother (two levels: no and yes).

### Data Analyses

For the purpose of analysis, the data were restructured so that each participant had a row for their father/stepfather; in total, 637 participants had multiple observations because they had both father and stepfather. Descriptive statistics for data in the long format are shown in Table 2.

In order to analyze all the response variables in the same model simultaneously, we applied path analysis (Kline, 2016). Residual covariances among all the outcome variables were included in the model since these outcomes were all proxies of investment and since they were measured from the same father-offspring dyads. Because both the birth father and the stepfather were included in the analyses for 637 respondents, the standard errors of the estimates were corrected for clustering within respondent. All the response variables were treated as continuous and were estimated with robust maximum likelihood estimation, correcting the standard errors using a sandwich estimator. This included the variables with ordinal scale owing to their 5-category scale (Rhemtulla et al., 2012). If any, biases of regression estimates and their standard errors are expected to be negligible in a sample as large as ours (Li, 2021). To handle missing data (see Table 2), we used multiple imputation prior to fitting the main models and followed the guidelines given by von Hippel (2020) for the number of imputed datasets needed. By accepting a 5% change in the standard error of the estimates, we imputed 30 datasets using Multiple Imputation by Chained Equations (MICE) (StataCorp, 2021; White et al., 2011). The imputed sample included 8326 observations. All statistical analyses were performed using Stata 16 (StataCorp, 2019).

We ran two separate models. First, we investigated whether there were differences between father types (i.e., birth father in relationship with mother, separated birth father and stepfather) in outcome variables. Second, using a sample including all separated fathers and stepfathers, we included childhood co-residence duration to assess its association with different outcome variables. We investigated childhood co-residence duration using separated fathers only since those fathers who were still in a relationship with the mother during the interview all had full childhood co-residence by definition. To help interpret and visualize the results, we calculated predictive margins from the regression models (Klein, 2014; Williams, 2012).

## Results

### Comparison between Father Types

**Fig. 1:**
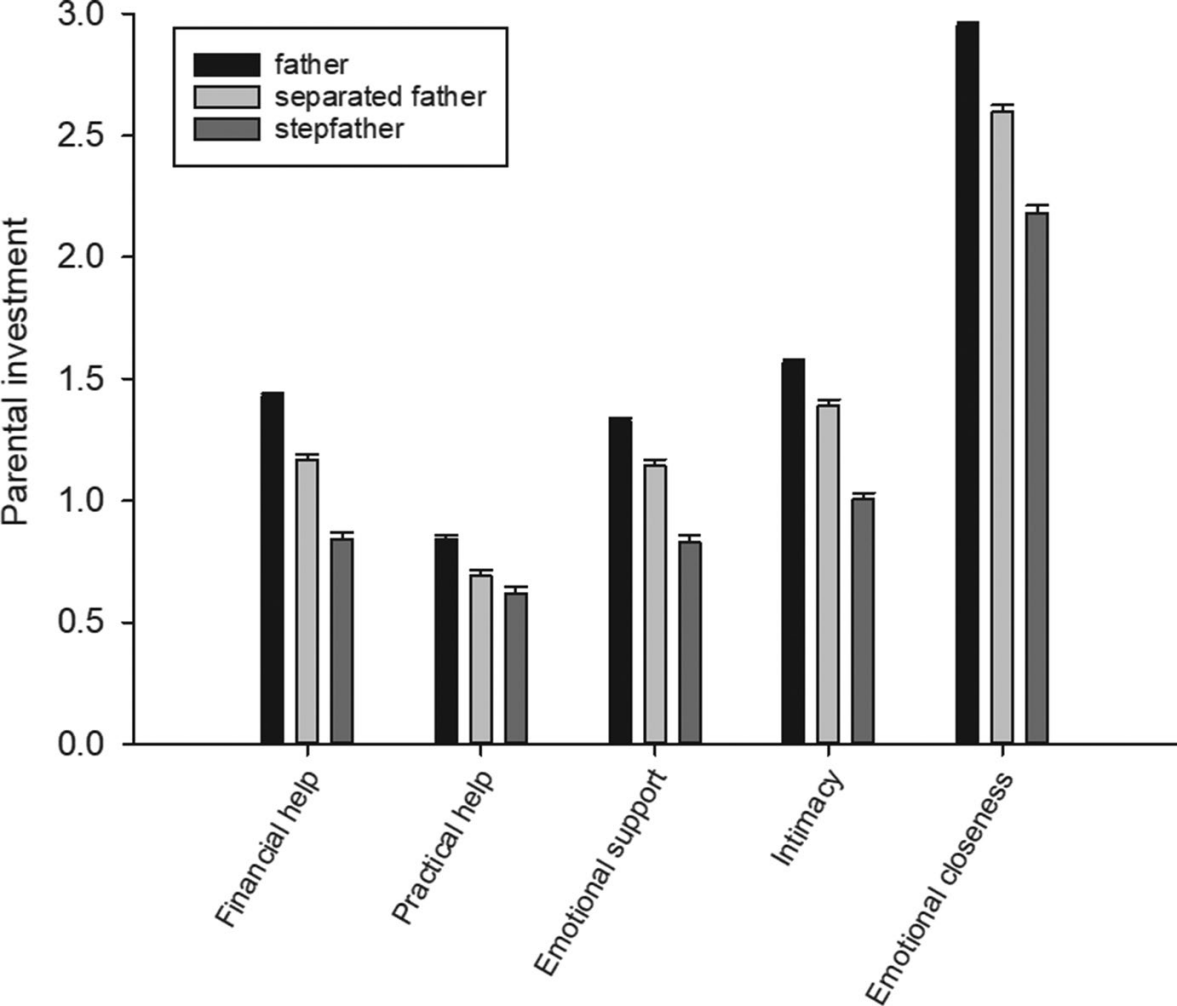
Marginal means and standard errors of investment of birth fathers who were still in a relationship with respondent’s mother (father), separated fathers, and stepfathers

We compared investment of three different father types: birth fathers currently in a relationship with the mother (“father”), separated birth fathers (“separated father”), and stepfathers currently in a relationship with the mother (“stepfather”). Fathers invested the most, and stepfathers the least. Results by father type are presented in Table 3; for full model results, see the Electronic Supplementary Material (ESM). Predicted means illustrate differences among father types (Fig. 1). The order of investment was similar in each investment measure. Overall, apart from closeness, younger cohorts received more investment than older cohorts (Table S1 in the ESM).

**Table 3:** Results for father type from model testing relationship between father type (birth father in relationship with mother as a reference) and different investment measures

	Coefficient	SE	*t*	*p*
Financial help				
separated father	− 0.263	0.029	− 9.210	< .0001
stepfather	− 0.588	0.030	− 19.450	< .0001
Practical help				
separated father	− 0.152	0.031	− 4.940	< .0001
stepfather	− 0.224	0.035	− 6.460	< .0001
Emotional support				
separated father	− 0.186	0.031	− 5.910	< .0001
stepfather	− 0.499	0.033	− 15.240	< .0001
Intimacy				
separated father	− 0.177	0.028	− 6.390	< .0001
stepfather	− 0.563	0.029	− 19.420	< .0001
Emotional closeness				
separated father	− 0.354	0.032	− 11.090	< .0001
stepfather	− 0.770	0.034	− 22.590	< .0001

### Childhood Co-residence Duration and Investment by Separated Fathers and Stepfathers

**Fig. 2:**
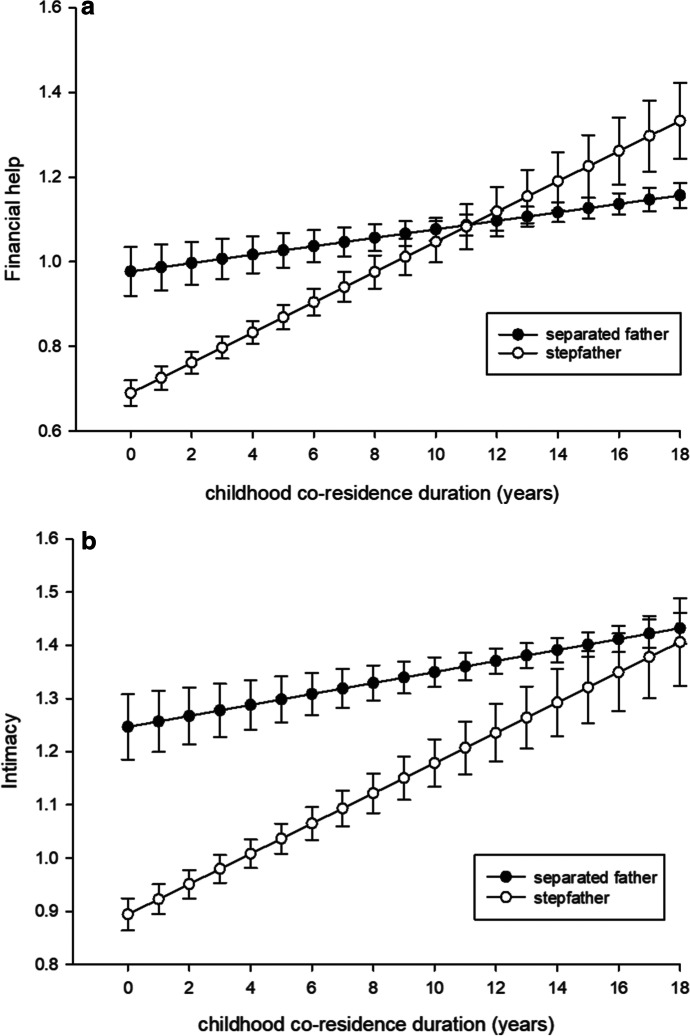
Model based predictive margins and standard errors for (**A**) financial help and (**B**) intimacy in relation to father type (separated father and stepfather) and childhood co-residence duration in years

For this analysis, we included only separated fathers and stepfathers to investigate how childhood co-residence duration was related to paternal investment in these two groups. For financial help and intimacy, there was a statistically significant interaction between childhood co-residence duration and father type (Table 4, for full model see ESM Table S2). To illustrate the interactions, Fig. 2 shows predicted margins for financial help and intimacy by childhood co-residence duration and by father type.

**Table 4:** Results for father type from model testing relationship between father type (separated father as a reference) and childhood co-residence duration and their interaction term

	Coefficient	SE	*t*	*P* >|*z*|
Financial help				
stepfather	− 0.287	0.067	− 4.310	< .0001
co-residence duration	0.010	0.004	2.550	0.01
interaction term	0.026	0.007	3.590	< .0001
Practical help				
stepfather	0.109	0.060	1.820	0.07
co-residence duration	0.015	0.004	4.150	< .0001
interaction term	0.003	0.008	0.460	0.646
Emotional support				
stepfather	− 0.210	0.073	− 2.880	0.004
co-residence duration	0.014	0.004	3.220	0.001
interaction term	0.012	0.008	1.610	0.108
Intimacy				
stepfather	− 0.353	0.069	− 5.130	< .0001
co-residence duration	0.010	0.004	2.480	0.013
interaction term	0.018	0.007	2.710	0.007
Emotional closeness				
stepfather	− 0.245	0.079	− 3.090	0.002
co-residence duration	0.020	0.005	4.160	< .0001
interaction term	0.006	0.008	0.730	0.470

Childhood co-residence duration was positively correlated with all investment measures (Table 4). To illustrate, Table 5 displays predictive margins at co-residence durations of 0, 3, 9, and 15 years.

**Table 5:** Relationship between childhood co-residence duration and investment of separated fathers and stepfathers at co-residence duration of 0, 3, 9, and 15 years. Predictive margins and standard errors are derived from the regression model

	Childhood co-residence duration (years)							
	0		3		9		15	
Separated fathers								
Financial help	0.98	0.06	1.007	0.05	1.07	0.029	1.13	0.02
Practical help	0.35	0.05	0.399	0.04	0.49	0.027	0.58	0.03
Emotional support	0.95	0.06	0.991	0.05	1.08	0.032	1.16	0.03
Intimacy	1.25	0.06	1.278	0.05	1.34	0.03	1.40	0.02
Emotional closeness	2.29	0.07	2.347	0.06	2.47	0.035	2.58	0.03
Stepfathers								
Financial help	0.69	0.03	0.797	0.03	1.01	0.044	1.23	0.07
Practical help	0.46	0.03	0.519	0.03	0.63	0.049	0.74	0.08
Emotional support	0.74	0.03	0.817	0.03	0.98	0.047	1.14	0.08
Intimacy	0.89	0.03	0.979	0.03	1.15	0.041	1.32	0.07
Emotional closeness	2.04	0.04	2.119	0.03	2.27	0.048	2.42	0.08

## Discussion

We investigated whether genetic relatedness and childhood co-residence duration are associated with paternal investment using cross-sectional survey data from Germany. In this survey, adolescent and adult children answered questions about their relationships with their birth and stepfathers. We predicted that birth fathers would invest more than stepfathers on the basis of inclusive fitness theory (Hamilton, 1964) and findings from prior studies (e.g., Anderson, 2012), and this was indeed the case. Even birth fathers who were separated from the respondent’s mother invested significantly more than did stepfathers. Kalmijn (2013), by contrast, reported that relationships with stepfathers were closer, on average, than those with separated fathers in a Dutch sample and explained this result as being mediated by the mother: Since children usually have a closer relationship with their mother than with their father, closeness to mother “exposes” stepchildren to a closer relationship with their stepfather.

Birth fathers who were still in a relationship with the mother invested more than those who were separated. This is unsurprising since many studies suggest that paternal effort is not solely an investment in the child but is also, at least in part, an investment in the father’s relationship with the mother (Anderson et al., 1999a; Rohwer et al., 1999). Although many men continue to invest in their children after separation or divorce, many “deadbeat dads” do not (e.g. Bartfeld & Meyer, 1994). We would expect that separation would have the effect of reducing investment by stepfathers even more than in birth fathers, since stepfathers get no “payback” in direct fitness from investment in a child. However, the relevant comparisons could not be made with the pairfam data, which does not ask about relationships with stepfathers whose relationship with the mother has ended. Overall, there has not been much research addressing the extent to which stepfathers maintain relationships with stepchildren and continue to invest in them after separating from the mother (Anderson, 2011a, b). It is certainly not unusual, however, for stepparent–stepchild relationships to be severed by such separations (Noël-Miller, 2013). For example, Bildtgård et al. (2021) reported on the basis of an interview study of Swedes who had raised both children and stepchildren that a relationship persisted only if some sort of positive connection between the parent and the former stepparent also persisted, and they conclude that relationships with birth parents and stepparents are qualitatively different because only the latter is a “mediated relationship.”

Childhood co-residence provides an opportunity to form a personal bond (Kalmijn, 2013), and we found that childhood co-residence duration was indeed associated with increased investment by both separated fathers and stepfathers. These results are in line with previous findings that the length of the parental investment period is related to the quality of (step)father–child relations (Daly et al., 1996; Hornstra et al., 2020; Kalmijn, 2013) and to financial support (Anderson et al., 1999a, b). It is worth noting, however, that residence with the child takes place in different periods of the childhood for the two father types: separated fathers resided with the child from birth until the age when the father departed, whereas for stepfathers co-residence usually began when the child was older, perhaps even an adult. One might assume that the years that the birth father spends with an infant and toddler would count for more than those that the stepfather spends with an older child, but our results indicate that childhood co-residence was positively correlated with investment in both father types.

Interestingly, interactions between father type and childhood co-residence duration in their effects on financial help and intimacy suggest that when childhood co-residence duration increases, investment by stepfathers approaches in magnitude that of the separated fathers. These results could also be interpreted to mean that for financial help and intimacy, childhood co-residence may not be as important for birth fathers as for stepfathers. An ultimate explanation is that childhood co-residence may have evolved to serve as a cue of genetic relatedness and thus promote kin altruism and investment (Lieberman et al., 2007; Tanskanen et al., 2021). Paternity certainty is often considered to be the most important factor affecting fathers’ parental investment (Queller, 1997), and males are expected to adjust their investment based on paternity confidence, or to balance costs of investing in others’ offspring with reproductive gains (Gray & Anderson, 2010). Results from the present study and others cited here suggest that co-residence during childhood increases parental investment. It is thus plausible that a close association with the child affects evolved psychological responses whose function is the identification of likely genetic offspring. More research on different societies is needed to know how universal this pattern may be. Huge variation in parental investment after divorce and remarriage exists between cultures; for example, in many Asian countries expectations of roles of noncustodial parents and stepparents reportedly differ in various ways from those in the modern West (Nozawa, 2020).

One strength of the present study is that we were able to investigate different forms of paternal investment: economic and practical help, and investment related to emotional support, closeness, and intimacy. Our approach differs from many previous social scientific studies by approaching parental investment from an evolutionary perspective, which treats parental investment, in any form (e.g., food and time), as a cost associated with raising an offspring, which reduces the parents’ ability to produce or invest in other current and future offspring and their own survival (Trivers, 1974). In this light, financial investment might be one of the clearest measures of parental investment because money used for the benefit of one offspring cannot be allocated elsewhere. For example, it has been suggested that paying child support to children from previous unions could potentially decrease the reproductive success of separated fathers, but the results are inconclusive because paying child support can also function as an honest signal of parenting and can be attractive to women (Anderson, 2011a, b). Korchmaros and Kenny (2001) proposed that emotional closeness mediates the effects of other variables on investment. In this study, emotional closeness was the outcome that had the highest values, and it followed the same pattern as other measures, with father rated closer than separated father and stepfathers rated last; closeness was also correlated positively with childhood co-residence duration in both father types.

A possible limitation of the study is that we have assumed that the childhood co-resident stepfather corresponds to the one at the time of interview, although mother’s partner might have changed. When comparing stepfathers with birth fathers, it is important to note that birth fathers resided with their children during the first years of life and, in this study population, paternity certainty is high—the extra-pair paternity rate is likely under one percent (Larmuseau et al., 2016; Wolf et al., 2012). Hence, birth fathers were presumably confident that the respondents were their biological offspring. Considering this, it is somewhat surprising that childhood co-residence duration was related to the amount of investment by separated birth fathers in emotional response variables (emotional support, intimacy, and emotional closeness), but of course, separation often involves discord and forces children to “choose” between their parents. Unfortunately, we could not control for (step)fathers’ socioeconomic status or possible non-resident children (biological or stepchildren), which can affect fathers’ ability to invest. Furthermore, we lack information on respondents’ siblings or half-siblings, which would be worth knowing because in a family in which the stepfather has biological children with the stepchild’s mother, investment in the stepchild may or may not equal investment in children of the new union (Hofferth & Anderson, 2003). This might affect our results if longer childhood co-residence duration is associated with having children of the new partnership.

Our findings support evolutionary inclusive fitness and mating effort theories in explaining social behavior and family dynamics. Furthermore, social environment, such as co-residence, tends to affect paternal investment. Cultural norms concerning fatherhood can vary between societies, and social expectations can be important factors affecting paternal investment. For example, among Himba pastoralists of Namibia, investing generously in genetically unrelated offspring can bring reputational benefits to men (Prall & Scelza, 2020). In Western countries, fatherhood has assumed a greater role in men’s life in recent decades, and to what extent this may also apply to stepfathers is an important question for further research (Gold & Edin, 2021). Our results suggest that co-residence during childhood is a potential bonding mechanism between stepfather and stepchild. The stepfather–stepchild relationship that starts when the child is young can take a different form than the relationship that starts when a child is an adolescent or an adult; acknowledging this could help interpret family dynamics better.

## Supplementary Information

Below is the link to the electronic supplementary material.

Supplementary file1 (PDF 227 KB)
