# Supplementary material for: Parental Investment by Birth Fathers and Stepfathers: Roles of Mating Effort and Childhood Co-residence Duration
Source: Hum Nat. 2023 Jun 10;34(2):276–94. doi: 10.1007/s12110-023-09450-6 (PMC10354157; doi:10.1007/s12110-023-09450-6)
Supplement: Supplementary file 1 — Supplementary file1 (PDF 227 KB) [file 12110_2023_9450_MOESM1_ESM.pdf]

Electronic Supplementary Material (ESM) for

Parental investment by birth fathers and stepfathers: roles of mating effort and childhood co-residence duration

Jenni E. Pettay[1*], Mirkka Danielsbacka[1 2], Samuli Helle[1], Gretchen Perry[3], Martin Daly[4], and Antti O. Tanskanen[1 2]

Human Nature 34(2), 2023, https://doi.org/10.1007/s12110-023-09450-6

[1] Department of Social Research, University of Turku, Turku, Finland

[2] Population Research Institute, Helsinki, Finland

[3]Department of Sociology, Anthropology & Human Services, University of Canterbury, Christchurch, New Zealand

[4]Department of Psychology, Neuroscience & Behaviour, McMaster University, Hamilton, ON, Canada

*corresponding author: Department of Social Research, Assistentinkatu 7, 20014, University of Turku, Turku, Finland. jenni.pettay@utu.fi

Table S1. Results regression analysis generalized structural equation model including residual covariances among all the outcome variables (Financial help, practical help, emotional support, intimacy, and emotional closeness). Total, imputed sample was 8326 observations of father, separated father and stepfather.

	Coefficient	std.err	t	P>|z|
**Financial help**				
father class (biological father with mother)				
biological father divorced	-0.2626	0.0285	-9.2100	<.0001
stepfather (with mother)	-0.5881	0.0302	19.4500	<.0001
Female	0.0984	0.0211	4.6500	<.0001
Cohort (1991-1993)				
1981-1983	-0.6412	0.0455	14.0900	<.0001
1971-1973	-0.9243	0.0530	17.4500	<.0001
Ethnicity (German native)				
Other countries	0.0475	0.0266	1.7900	0.07
Maternal education (lower level education)				
Higher level education	0.1620	0.0289	5.6000	<.0001
Respondent lives with mother	-0.0009	0.0429	-0.0200	0.98
Relationship status (not cohabiting)				
Cohabiting	-0.1307	0.0321	-4.0800	<.0001
Respondent's education (continuous)	0.0155	0.0114	1.3600	0.173
Children (none=0)				
at least one child	-0.0874	0.0318	-2.7500	0.006
travel time from respondent dwelling to step(fathers) house (continuous)	-0.0564	0.0079	-7.1800	<.0001
Constant	1.9008	0.0472	40.2600	<.0001
**Practical help**				
father class (biological father with mother)	-0.1523	0.0308	-4.9400	<.0001
biological father divorced	-0.2237	0.0346	-6.4600	<.0001
Female	0.0120	0.0252	0.4800	0.634
Cohort (1991-1993)				
1981-1983	-0.1921	0.0571	-3.3700	0.001
1971-1973	-0.1812	0.0666	-2.7200	0.007
Ethnicity (German native)				
Other countries	0.0459	0.0311	1.4800	0.140
Maternal education (lower level education)				
Higher level education	-0.0561	0.0332	-1.6900	0.091
Respondent lives with mother	-0.2312	0.0537	-4.3100	<.0001
Relationship status (not cohabiting)				
Cohabiting	-0.0381	0.0355	-1.0700	0.284
Respondent's education (continuous)	0.0458	0.0136	3.3700	0.001
Children (none=0)				

at least one child	0.0105	0.0375	0.2800	0.779
travel time from respondent dwelling to step(fathers) house (continuous)	-0.1648	0.0084	19.5800	<.0001
Constant	1.2369	0.0599	20.6500	<.0001
**Emotional support**				
father class (biological father with mother)				
biological father divorced	-0.1859	0.0314	-5.9100	<.0001
stepfather (with mother)	-0.4992	0.0328	15.2400	<.0001
Female	0.2420	0.0227	10.6500	<.0001
Cohort (1991-1993)				
1981-1983	-0.0947	0.0481	-1.9700	0.049
1971-1973	-0.1981	0.0578	-3.4300	0.001
Ethnicity (German native)				
Other countries	-0.0482	0.0287	-1.6800	0.094
Maternal education (lower level education)				
Higher level education	0.1052	0.0305	3.4500	0.001
Respondent lives with mother	-0.0593	0.0454	-1.3100	0.191
Relationship status (not cohabiting)				
Cohabiting	0.0170	0.0357	0.4800	0.634
Respondent's education (continuous)	0.0169	0.0124	1.3600	0.173
Children (none=0)				
at least one child	-0.0963	0.0371	-2.5900	0.010
**travel time from respondent dwelling to step(fathers) house** (continuous)	-0.0121	0.0087	-1.4000	0.162
Constant	1.3099	0.0508	25.7800	<.0001
**Intimacy**				
father class (biological father with mother)				
biological father divorced	-0.1772	0.0277	-6.3900	<.0001
stepfather (with mother)	-0.5630	0.0290	19.4200	<.0001
Female	0.0928	0.0198	4.6800	<.0001
Cohort (1991-1993)				
1981-1983	-0.1003	0.0434	-2.3100	0.021
1971-1973	-0.2267	0.0512	-4.4300	<.0001
Ethnicity (German native)				
Other countries	-0.0593	0.0255	-2.3200	0.020
Maternal education (lower level education)				
Higher level education	0.0633	0.0263	2.4000	0.016
Respondent lives with mother	-0.0498	0.0414	-1.2000	0.230
Relationship status (not cohabiting)				
Cohabiting	0.1005	0.0305	3.2900	0.001
Respondent's education (continuous)	0.0043	0.0110	0.3900	0.699
Children (none=0)				
at least one child	-0.1028	0.0317	-3.2400	0.001

travel time from respondent dwelling to step(fathers) house (continuous)	-0.0203	0.0077	-2.6300	0.008
Constant	1.6490	0.0459	35.9400	<.0001
**Emotional closeness**				
father class (biological father with mother)				
biological father divorced	-0.3536	0.0319	11.0900	<.0001
stepfather (with mother)	-0.7700	0.0341	22.5900	<.0001
Female	-0.0166	0.0218	-0.7600	0.446
Cohort (1991-1993)				
1981-1983	-0.0159	0.0454	-0.3500	0.726
1971-1973	-0.0881	0.0559	-1.5800	0.115
Ethnicity (German native)	0.0008	0.0277	0.0300	0.978
Other countries				
Maternal education (lower level education)				
Higher level education	-0.0435	0.0290	-1.5000	0.134
Respondent lives with mother	-0.1894	0.0435	-4.3600	<.0001
Relationship status (not cohabiting)				
Cohabiting	0.0838	0.0345	2.4300	0.015
Respondent's education (continuous)	-0.0523	0.0120	-4.3400	<.0001
Children (none=0)				
at least one child	-0.0801	0.0357	-2.2500	0.025
travel time from respondent dwelling to step(fathers) house (continuous)	-0.0789	0.0088	-8.9600	<.0001
Constant	3.2909	0.0485	67.9200	<.0001
var(e.financial)|	0.8436	0.0134		
var(e.practical)|	1.1886	0.0208		
var(e.support)|	0.9798	0.0146		
var(e.intimacy)|	0.7672	0.0116		
var(e.closeness)|	0.9464	0.0138		
cov(e.financial,e.practical)|	0.1796	0.0125	14.4200	<.0001
cov(e.financial,e.support)|	0.2238	0.0110	20.3100	<.0001
cov(e.financial,e.intimacy)|	0.1737	0.0095	18.3700	<.0001
cov(e.financial,e.closeness)|	0.1619	0.0101	15.9800	<.0001
cov(e.practical,e.support)|	0.2081	0.0137	15.1700	<.0001
cov(e.practical,e.intimacy)|	0.1395	0.0116	12.0700	<.0001
cov(e.practical,e.closeness)|	0.1416	0.0115	12.3400	<.0001
cov(e.support,e.intimacy)|	0.5593	0.0115	48.7100	<.0001
cov(e.support,e.closeness)|	0.3791	0.0111	34.2400	<.0001
cov(e.intimacy,e.closeness)|	0.4701	0.0101	46.5500	<.0001

Table S2. Results regression analysis generalized structural equation model including residual covariances among all the outcome variables (Financial help, practical help, emotional support, intimacy, and emotional closeness). Total, imputed sample was 2906 observations of separated father and stepfather.

	Coefficient	std.err	t	P>|z|
**Financial help**				
fatherclass (separated father)				
Stepfather	-0.287	0.067	-4.310	<.0001
childhood co-residence				
duration	0.010	0.004	2.550	0.011
fatherclass*childhood co-residence				
duration (stepfather)	0.026	0.007	3.590	<.0001
Female	0.030	0.037	0.800	0.426
Cohort (1991-1993)				
1981-1983	-0.538	0.076	-7.060	0.000
1971-1973	-0.698	0.087	-8.000	0.000
Ethnicity (German native)				
Other countries	0.081	0.052	1.570	0.116
Maternal education (lower level education)				
Higher level education	0.072	0.050	1.440	0.151
Respondent lives with mother	-0.099	0.064	-1.550	0.121
Relationship status (not cohabiting)				
Cohabiting	-0.151	0.050	-3.040	0.002
Respondent's education (continuous)	-0.006	0.020	-0.280	0.782
Children (none=0)				
at least one child	-0.103	0.052	-1.990	0.046
travel time from respondent dwelling to step(fathers) house (continuous)	-0.049	0.012	-4.070	<.0001
Constant	1.523	0.093	16.430	<.0001
**Practical help**				
fatherclass (divorced father)				
Stepfather	0.109	0.060	1.820	0.069
childhood co-residence				
duration	0.015	0.004	4.150	<.0001
fatherclass*childhood co-residence				
duration (stepfather)	0.003	0.008	0.460	0.646
Female	0.015	0.039	0.390	0.699
Cohort (1991-1993)				
1981-1983	-0.277	0.092	-3.000	0.003
1971-1973	-0.224	0.102	-2.190	0.028
Ethnicity (German native)				
Other countries	0.037	0.052	0.720	0.473

Maternal education (lower level education)				
Higher level education	-0.019	0.050	-0.370	0.711
Respondent lives with mother	-0.329	0.079	-4.180	0.000
Relationship status (not cohabiting)				
Cohabiting	-0.197	0.049	-4.030	0.000
Respondent's education (continuous)	0.004	0.021	0.180	0.861
Children (none=0)				
at least one child	-0.015	0.052	-0.280	0.782
travel time from respondent dwelling to step(fathers) house (continuous)	-0.128	0.014	-9.340	<.0001
Constant	0.984	0.099	9.910	<.0001
**Emotional support**				
fatherclass (separated father)				
Stepfather	-0.210	0.073	-2.880	0.004
childhood co-residence				
duration	0.014	0.004	3.220	0.001
fatherclass*childhood co-residence				
duration (stepfather)	0.012	0.008	1.610	0.108
Female	0.167	0.041	4.100	<.0001
Cohort (1991-1993)				
1981-1983	-0.156	0.083	-1.880	0.060
1971-1973	-0.243	0.098	-2.480	0.013
Ethnicity (German native)				
Other countries	-0.068	0.057	-1.210	0.227
Maternal education (lower level education)				
Higher level education	0.101	0.055	1.830	0.068
Respondent lives with mother	-0.256	0.070	-3.650	<.0001
Relationship status (not cohabiting)				
Cohabiting	-0.046	0.058	-0.800	0.424
Respondent's education (continuous)	-0.002	0.022	-0.080	0.935
Children (none=0)				
at least one child	-0.098	0.063	-1.570	0.117
travel time from respondent dwelling to step(fathers) house (continuous)	-0.026	0.013	-1.920	0.054
Constant	1.170	0.105	11.130	<.0001
**Intimacy**				
fatherclass (separated father)				
Stepfather	-0.353	0.069	-5.130	<.0001
childhood co-residence				
duration	0.010	0.004	2.480	0.013
fatherclass*childhood co-residence				
duration (stepfather)	0.018	0.007	2.710	0.007
Female	0.027	0.036	0.740	0.459

Cohort (1991-1993)				
1981-1983	-0.165	0.074	-2.220	0.026
1971-1973	-0.319	0.086	-3.730	<.0001
Ethnicity (German native)				
Other countries	-0.093	0.050	-1.860	0.063
Maternal education (lower level education)				
Higher level education	0.066	0.051	1.290	0.196
Respondent lives with mother	-0.225	0.065	-3.440	0.001
Relationship status (not cohabiting)				
Cohabiting	0.054	0.048	1.110	0.268
Respondent's education (continuous)	-0.010	0.020	-0.500	0.619
Children (none=0)				
at least one child	-0.067	0.055	-1.220	0.221
travel time from respondent dwelling to step(fathers) house (continuous)	-0.043	0.012	-3.560	<.0001
Constant	1.573	0.098	16.080	<.0001
**Emotional closeness**				
fatherclass (separated father)				
Stepfather	-0.245	0.079	-3.090	0.002
childhood co-residence				
duration	0.020	0.005	4.160	<.0001
fatherclass*childhood co-residence				
duration (stepfather)	0.006	0.008	0.730	0.466
Female	-0.108	0.041	-2.650	0.008
Cohort (1991-1993)				
1981-1983	-0.055	0.080	-0.680	0.495
1971-1973	-0.066	0.097	-0.670	0.500
Ethnicity (German native)				
Other countries	-0.080	0.056	-1.430	0.151
Maternal education (lower level education)				
Higher level education	-0.081	0.056	-1.440	0.149
Respondent lives with mother	-0.331	0.067	-4.910	<.0001
Relationship status (not cohabiting)				
Cohabiting	0.051	0.057	0.900	0.368
Respondent's education (continuous)	-0.083	0.023	-3.670	<.0001
Children (none=0)				
at least one child	-0.131	0.063	-2.090	0.037
travel time from respondent dwelling to step(fathers) house (continuous)	-0.096	0.014	-7.090	<.0001
Constant	2.926	0.104	28.130	<.0001
var(e.financial)|	0.844	0.024		
var(e.practical)|	0.928	0.036		
var(e.support)|	1.029	0.027		

var(e.intimacy)|	0.835	0.022		
var(e.closeness)|	1.131	0.025		
cov(e.financial,e.practical)|	0.223	0.021	10.850	<.0001
cov(e.financial,e.support)|	0.316	0.020	15.620	<.0001
cov(e.financial,e.intimacy)|	0.253	0.017	14.670	<.0001
cov(e.financial,e.closeness)|	0.257	0.019	13.780	<.0001
cov(e.practical,e.support)|	0.279	0.023	12.190	<.0001
cov(e.practical,e.intimacy)|	0.212	0.019	10.920	<.0001
cov(e.practical,e.closeness)|	0.208	0.020	10.600	<.0001
cov(e.support,e.intimacy)|	0.643	0.022	29.550	<.0001
cov(e.support,e.closeness)|	0.493	0.021	23.190	<.0001
cov(e.intimacy,e.closeness)|	0.589	0.019	30.460	<.0001
